# Current Use of Fenton Reaction in Drugs and Food

**DOI:** 10.3390/molecules27175451

**Published:** 2022-08-25

**Authors:** Chizumi Abe, Taiki Miyazawa, Teruo Miyazawa

**Affiliations:** New Industry Creation Hatchery Center (NICHe), Tohoku University, Sendai 980-8579, Japan

**Keywords:** antioxidants, cancer, Fenton reaction, hydrogen peroxide, hygiene, iron, nanomedicine, oxidative stress, polyphenol, vitamin C

## Abstract

Iron is the most abundant mineral in the human body and plays essential roles in sustaining life, such as the transport of oxygen to systemic organs. The Fenton reaction is the reaction between iron and hydrogen peroxide, generating hydroxyl radical, which is highly reactive and highly toxic to living cells. “Ferroptosis”, a programmed cell death in which the Fenton reaction is closely involved, has recently received much attention. Furthermore, various applications of the Fenton reaction have been reported in the medical and nutritional fields, such as cancer treatment or sterilization. Here, this review summarizes the recent growing interest in the usefulness of iron and its biological relevance through basic and practical information of the Fenton reaction and recent reports.

## 1. Introduction

It has only been around 100 years since people started research on food in terms of “modern nutritional science” [1]. In the latter part of the 1800s, Lavoisier investigated chemical oxidation in living things, which is thought to be the beginning of nutritional science. In the early 1900s, individual food components began to be found. For example, vitamin B1 (oryzanin) was found in trials to overcome the deficiencies of military patients [2]. Vitamin E was isolated from wheat germ oil, which was found to be involved in rat reproduction [3]. In contrast, the discovery of minerals dates back to 6000 B.C, but it took centuries to recognize their roles in biological systems. Among the various minerals, iron is one of the well-known and -utilized minerals from ancient times and was estimated to be present in the blood in the 1700s [4]. Iron is the most abundant transition metal on Earth’s surface, with 3–4 g of iron in the body of a healthy adult human [5,6]. Since iron is the most abundant transition metal in the human body, its contribution toward various biological activities has long been the focus of growing attention [7]. For example, anemia is one of the major manifestations of iron deficiency. In 1925, Fontès and Thivolle found that iron-deficient horses had lower serum iron concentrations [8]. In the human body, most extracellular iron is bound to iron-binding proteins (such as transferrin and lactoferrin) [9]. Heme proteins in red blood cells play an important role in transporting oxygen to organs [10].

On the other hand, iron is involved not only in the delivery of oxygen in our body but also in DNA synthesis and/or repair [11], indicating this mineral is essential for the survival of living things. Iron also works as a cofactor to facilitate various enzymes, such as catalase and cytochromes. The roles of iron in the body are particularly involved in redox reactions due to its preferable affinity to oxygen. In the 1890s, Henry John Horstman Fenton found the redox reaction between iron (II) and hydrogen peroxide (H_2_O_2_) to produce hydroxyl radical (OH^•^), called the Fenton reaction [12]. This reaction potentially occurs in the human body and is thought to regulate complicated systems, which is related to homeostasis. The products of the Fenton reaction OH^•^ is highly reactive particles that induce oxidative damage to cells, but this is also an aspect that can be a therapeutic strategy for cancer patients. Several medicines (e.g., doxorubicin (DOX) [13], β-lapachone [14], and cisplatin [15]) include mechanisms of reaction that have applied the Fenton reaction to generate the poison, OH^•^, to cancer cells. Additionally, numerous food components daily consumed have beneficial effects in the human body, such as on chronic diseases and on immune systems [16,17,18]. The antioxidant reaction is one of the major properties of such food components (e.g., polyphenols and vitamins). Considering reactive oxygen species (ROS) generated by vitamin C [19] and chelating metal iron by flavonoids [20], iron potentially affects the bioactivities of absorbed and metabolized food components in the body. Furthermore, there is growing interest in programed death “ferroptosis” related to the Fenton reaction [21]. Against these backgrounds, this review summarizes the recent growing interest in the usefulness of iron and its biological relevance through basic and practical information of the Fenton reaction and recent reports.

## 2. Fenton Reaction

The Fenton reaction is the reaction of iron (II) with H_2_O_2_, reported by Henry John Horstman Fenton in 1894 [12]. In 1876, his student found that a mixture of H_2_O_2_, tartaric acid, ferrous salt and water turned a violet color. This is known as the Fenton reaction (Reaction (1)):

Fe^2+^ + H_2_O_2_ → Fe^3+^ + OH^−^ + OH^•^ (1)

While Fenton speculated the mechanism of oxidation by H_2_O_2_ and iron (II), some researchers doubted the formation of OH^•^ in one-electron reduction by iron (II). In 1931, Haber and Wilstatter mentioned the hydroxy radical in radical chain mechanisms (Reaction (2) and (3)) [22]. They described that chain reactions are initiated by enzymes, specifically catalase:

OH + H_2_O_2_ → H_2_O + O_2_H (2)

O_2_H + H_2_O_2_ → O_2_ + H_2_O + OH (3)

Thereafter, Harber and Weiss explained the decomposition of H_2_O_2_ by iron (II) using Reaction (4) to (6), where the Fenton reaction initiates and Reaction (6) terminates the chain reactions [23]:

OH^•^ + H_2_O_2_ → H_2_O + O_2_^•−^ + H^+^ (4)

O_2_^•−^ + H^+^ + H_2_O_2_ → O_2_ + H_2_O + OH^•^ (5)

Fe^2+^ + HO + H^+^ → Fe^3+^ + H_2_O (6)

In 1937, Weiss explained the reaction mechanism of catalase: an anion H_2_O_2_ reduces iron (III) to iron (II), and then iron (II) reduces H_2_O_2_ to OH^•^ and water, followed by chain reaction (5, 6), which is collectively referred to as the Haber–Weiss reaction. The mechanism of the Fenton reaction has studied and discussed, among which detailed equilibrium principles have been well summarized by Stanbury [24]. The Fenton reaction is affected by the environmental pH and concentration of iron. The major ROS generated from the Fenton reaction are oxoiron (IV) species at pH > 3, and OH^•^ at more acidic conditions [25,26,27]. Two mechanisms, the “radical mechanism” and the “complex mechanism”, contribute to the iron-catalyzed disproportionation of H_2_O_2_ and the Fenton reaction. The products obtained from these reactions are different. In the “radical mechanism”, Fe^2+^ and Fe^3+^ react with H_2_O_2_ to produce OH^•^ and superoxide, respectively. In the “complex mechanism”, Fe^2+^ and Fe^3+^ react with H_2_O_2_ to produce FeO^2+^ and FeO^3 +^, respectively. In 2013, more than 100 years after the Fenton reaction was proposed, successful detection of Fe(IV) was reported [28]. Additionally, it has been suggested that fellyl ion controls iron cycling by the Fenton reaction in a cloud as well as Fe^2+^ and Fe^3+^ [29]. Such reports indicate how great the impact and complexity of this reaction is. The use of other transition metals such as copper leads to a reaction similar to the Fenton reaction, called the Fenton-like reaction. Although the Fenton reaction initially began to be used for analytical purposes, chelation or sequestration of transition metals involving Fenton and Fenton-like reactions have been found to play important roles in the internal and external environments of living things.

## 3. Fenton Reaction in Body

### 3.1. Iron as a Nutrient

Nutrients are essential for living things, among which proteins, fats, and carbohydrates are three major nutrients. In the context of the human diet, minerals are elements, except H, C, N, and O (the main components of three major nutrients: organic compounds), that maintain or regulate biological systems, and account for approximately 4% of the human body. Of these, 16 types of elements (Na, N, P, K, S, Ca, Mg, iodine, Se, Cr, Co, Fe, Mn, Zn, Cu, and Mo) are thought to play particularly important roles. They are classified into two groups based on the required amount (more than 100 mg/day: Na, N, P, K, S, Ca, and Mg; less than 100 mg/day: iodine, Se, Cr, Co, Fe, Mn, Zn, Cu, and Mo).

**Figure 1:**
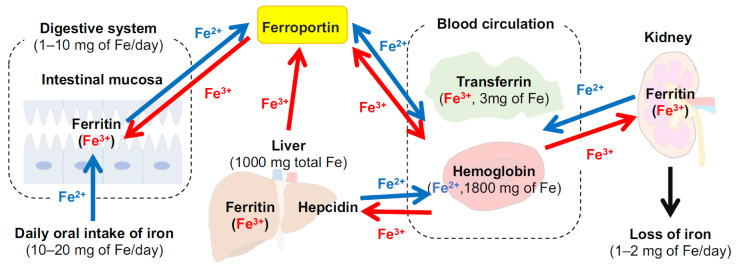
Distribution of iron in the body and the main organs involved in the regulation of iron metabolism (modified from permission from [39] under the Creative Commons CC BY 4.0 license, https://creativecommons.org/licenses/by/4.0/ (accessed on 28 July 2022)). Values data for iron levels are obtained from Lesjac et al. [40]).

Iron is present in all human cells, with an average of 2.4 g in women and 3.8 g in men, with daily losses of 1–2 mg [30] (Figure 1). Examples of iron-rich foods include oysters, clams, mussels, beef or chicken liver, and poultry while non-heme iron is contained in beans, spinach, nuts, and seeds. One of the most important roles of iron is to transport oxygen in hemoglobin (Hb). This protein, consisting of 96% of blood cells [31], provides oxygen to the whole body from the lungs or other airway organs and supports metabolism. Hb iron binds up to four oxygen molecules in the form of Fe^2+^ or Fe^3+^ [32]. Additionally, other oxygen storage protein and enzymes bind to iron (hemoglobin, 2500 mg iron; myoglobin, 130 mg iron; enzymes, 150 mg iron) [33]. Anemia due to a general iron deficiency (Hb <13 g/dL in males, <12 g/dL in females, <11 g/dL during pregnancy) is mainly due to biological mechanisms (e.g., iron deficiency, hemolytic anemia, and anemia of inflammation) and/or erythrocyte morphology. Iron deficiency occurs when there is an insufficient supply of iron against the amount needed such as during periods of high iron requirements (e.g., infancy and pregnancy) and/or iron loss exceeds intake. Iron is absorbed via human intestinal mucosa in heme and non-heme forms, whereas heme-iron is reported to be more readily absorbed through the folate transporter [34]. Non-heme iron, Fe^2+^ and Fe^3 +^, is transported into the duodenal cytoplasm via divalent metal iron transporter-1 (DMT-1) in Fe^2+^ [35], where Fe^3+^ is previously reduced to Fe^2+^ by cytochrome b reductase and/or other reductants. After being transported into cells, iron either binds to ferritin for storage or is transported into the blood stream via ferroportin as Fe^2 +^. Iron is then oxidized by membrane-bound ferroxidase hephaestin and ceruloplasmin to be incorporated into transferrin to form the transferrin–Fe^3+^ complex. Hepcidin is a peptide hormone excreted from the liver that binds to ferroportin, the only iron efflux transporter in the blood, and regulates iron homeostasis by promoting internalization and degradation of the transporter [36]. Hepcidin completely occludes the iron pathway by binding ferroportin with an outward-open conformation [37]. While this section only presented limited information on iron absorption and metabolism, more detailed clinical characteristics of iron deficiency are described by Camaschella et al. and Pasricha et al. [33,38].

**Figure 2:**
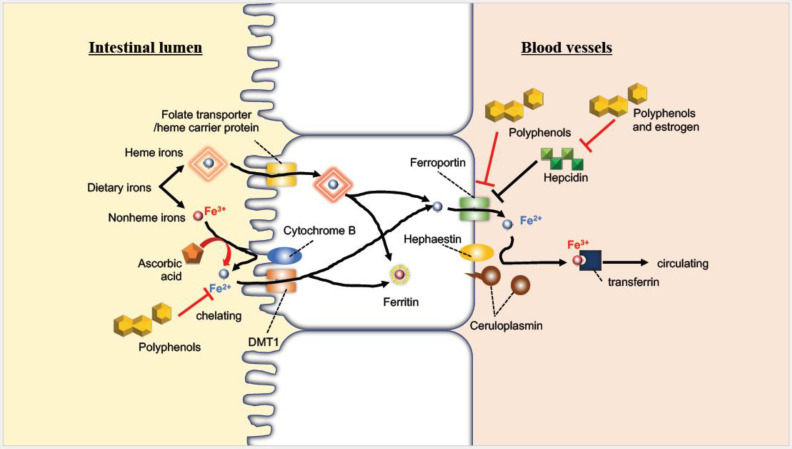
Absorption behavior of iron in the intestine and the interaction with molecules derived from food (modified with permission from [50] under the Creative Commons CC BY 4.0 license, https://creativecommons.org/licenses/by/4.0/ (accessed on 28 July 2022)).

As mentioned above, iron is absorbed in the intestine as either heme or non-heme forms, but other food-derived components are also absorbed in the intestine, suggesting that interactions with them may affect iron absorption (Figure 2). For example, quercetin has been reported to inhibit intestinal iron absorption by different mechanisms, through chelation in an acute duodenal injection study and by suppressing ferroportin expression in an oral administration study in rats [41]. Tea consumption also reduces the bioavailability of iron, possibly due to polyphenols such as tannins [42,43]. It has also been reported that the intake of a high-fat diet inhibits intestinal iron absorption, causing iron deficiency [44], and that the amount of absorbed iron in overweight women was two-thirds of the normal value [45]. In contrast, ascorbic acid is well known to increase iron absorption related to iron reduction and the intake of ascorbic acid attenuates the above inhibitory effect of polyphenols [46]. The major peptide hormone, hepcidin, is also affected by flavonoids; myricetin significantly suppresses the expression of this hormone [47]. Higher concentrations and lower clearance of hepcidin due to chronic kidney disease suppress iron absorption, resulting in iron deficiency [48]. On the contrary, 17β-estradiol possibly promotes iron absorption by inhibiting hepcidin expression through an estrogen-responsive element half-site in the promoter region of the hepcidin gene [49], indicating that increased iron might be caused by another mechanism in postmenopausal women.

### 3.2. Fenton Reaction under Biological Environment

**Figure 3:**
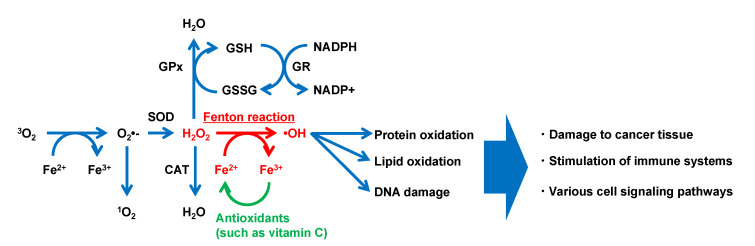
Typical model of reactive oxygen species generation via the Fenton reaction in a biological environment. CAT, catalase; GPx, glutathione peroxidase; GR, glutathione reductase; GSH, reduced glutathione; GSSG, oxidized glutathione; NADPH, reduced form of nicotinamide adenine dinucleotide phosphate; NADP ^+^, oxidized form of nicotinamide adenine dinucleotide phosphate; SOD, superoxide dismutase (modified with permission from [61] under the Creative Commons CC BY 3.0 license, https://creativecommons.org/licenses/by/3.0/ (accessed on 28 July 2022)).

Iron is transferred from binding in transferrin as Fe^3+^ form and transported into cells via the transferrin receptor. Most transition metals, including iron, are involved in the generation of various free radicals due to their redox features. As “second-messengers”, ROS play essential roles in cellular life cycles, such as proliferation [51] and gene expression [52] (Figure 3). Observation with fluorescent reagents (e.g., dihydrorhodamine, coumarin-3-calboxylic acid, and endoplasmic reticulum (ER)-targeting OH^•^ probe) has revealed intracellular localization of the generated OH^•^. Such studies have reported that ER by the Fenton reaction [53,54] regulates hypoxia-inducible gene expression. ER stress has been reported to regulate more than one-third of all proteins made in the cell in synthesis, folding, and structural maturation [55]. Additionally, H_2_O_2_, one type of ROS generated extracellularly, penetrates the cell membrane easily to react with intracellular iron (Fe^2+^ and Fe^3 +^), producing OH^•^ through the Fenton and Fenton-like reaction. OH^•^ reacts most strongly with biomolecules shorter than 1 ns [3,56], which results in the most severe damage to biological systems among ROS. It involves the induction of the oxidation of molecules. OH^•^ produced through the Fenton reaction has been reported to induce DNA damage [57]. Iron released from Hb is also known to promote the degradation of deoxyribose, inducing lipid peroxidation [58,59]. Additionally, Fenton-type chemistry (e.g., peroxidases, free heme, and metal ions) is involved in the tyrosine nitration observed within tyrosine residues in proteins and used as a signature for peroxynitrite [60].

In heme proteins, the transition of iron is essential for the performance of their functions. Among them, cytochrome P450 is one of the largest enzyme families, in which as many as 18,000 P450s have been identified [62] and is well known to work in detoxification of drugs or other xenobiotics. This enzyme is made up of 40–50 kDa single polypeptides with a long I helix and H-bond between Cys, and a peptide NH group is regarded as the key factor to heme iron redox. Fe^2+^ centered in the enzyme binds to O_2_ to form oxy complex followed by the second electron transfer and heterolytic cleavage, during which ROS can be produced. Heme degradation catalyzed by heme oxygenases also generates ROS by non-heme iron.

Recently, ferritinophagy and ferroptosis have attracted attention as iron-dependent cell death. Ferritinophagy consists of the autophagic degradation of ferritin to regulate iron homeostasis [63]. Increased intracellular iron levels following the release from ferritin promotes ROS production, leading to cell death; radiation is reported to induce autophagic iron-dependent death in cancer cells, which is a promising therapeutic strategy [64]. “Ferroptosis”, coined by Brent Roark Stockwell and Scott Dixon in 2012 [65], is one type of regulated cell death dependent on iron or ROS, which is distinct from other types such as apoptosis, necrosis, and autophagic death at the morphological, biochemical, and genetic levels. Excessive iron in cell lines (harboring RAS mutations with increased iron uptake and decreased iron storage) induces ferroptosis, which is regulated by suppression of the master transcription factor of iron metabolism [66], indicating that ferroptosis is iron dependent. Although the correlation between autophagy and ferroptosis is not well understood, Park et al. elucidated that ROS-induced autophagy plays an important role in ferritin degradation and transferrin receptor 1 expression during ferroptosis [67]. More details on ferroptosis are beyond the scope of this review and are reviewed and described by Xie et al. [68], Chen et al. [69], and Bebber et al. [70].

## 4. Use of the Fenton Reaction for Drugs

**Figure 4:**
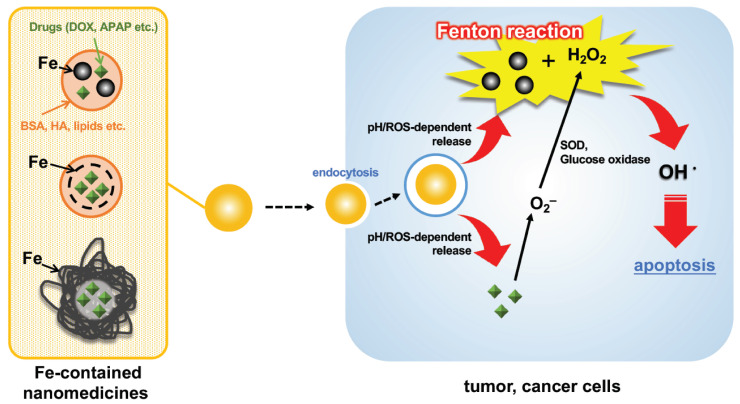
Depicted representative scheme of cell apoptosis by Fe-containing nanomedicines via the Fenton reaction. DOX, doxorubicin; APAP, amionoacetophen; BSA, bovine serum albumin; HA, hyaluronic acid; SOD, superoxide dismutase; ROS, reactive oxygen species.

ROS are regarded to cause intracellular lipid peroxidation, leading to ferroptosis. Therefore, the Fenton reaction has been challenged for use in directly attacking cancer cells, but it is difficult to treat them because of the low amounts of generated OH^•^ [71]. In recent years, various nanoparticles that enhance the effectiveness of Fenton reactions for drug applications (nanomedicines) have been reported. A simple scheme is depicted in Figure 4. Previous reports on such an approach have already been well reviewed by Meng et al. [72], Ranji-Burachaloo et al. [73], and Miyazawa et al. [74], so the present review focuses on very recent reports (from 2020) on nanomedicines using the Fenton reaction.

Xing et al. prepared an iron-loaded liposome using hollow mesoporous Prussian blue co-delivering iron, unsaturated lipids, and a photothermal converter. Controlled passive targeting enabled efficient photothermal effects and ferroptosis of these liposomes with low toxicity [75]. Tian et al. prepared ultra-small ellagic acid-Fe-bovine serum albumin nanoparticles and showed acceleration of Fe^3+^/Fe^2+^ transformation by strong reduction of endogenous H_2_S [76]. Sang et al. first prepared PZIF-67 nanoparticles with SOD (super oxide dismutase)-like activity and an OH -generating ability [77]. Gao et al. prepared the nanoparticles encapsulating light-responsive CO prodrugs by self-assembly of photoresponsive polymers. These nanoparticles accumulated in mitochondria and light-responsively released CO and the prodrugs, followed by the Fenton reaction, which generated high levels of ROS to decrease cell viability. Actually, intravenous injection of the nanoparticle significantly suppressed the tumor growth with an increase in ROS [78]. You et al. combined NIR irradiation with nanoparticles. Functional nanoparticles with internally encapsulated functional benzothiazole complexes (eTB2) and the photosensitizer indocyanine green induced FeTB2 release and Fenton reaction under NIR irradiation [79]. Wu et al. prepared hollow porous carbon coated with FeS_2_-based nanoparticles. Prepared hollow porous carbon revealed that the conversion of NIR heat into an effective temperature rise by the carbon shell and the reduction of Fe^3+^ to Fe^2+^ by tannic acid promoted the Fenton reaction [80]. Fu et al. reported that DOX and glucose oxidase-gallic acid/iron complexes were encapsulated into zeolitic imidazole framework-8 nano particles, which induced cancer cell death by the Fenton reaction with gallic acid/iron complexes under an acidic microenvironment [81]. Chen et al. reported the preparation of biodegradable nanoparticles of Fe_3_O_4_ bound with protocatechuic acid and human serum albumin loaded with β-lapachone [82].

Correlating pH or ROS sensitivity with the Fenton reaction enables more effective and selective attack of tumor cells, which is also being used as an approach. Sun et al. prepared synergistically therapeutic nanoparticles that encapsulated acetaminophen (APAP). Sun et al. prepared nanoparticles with the ability to induce the Fenton reaction in a weakly acidic tumor microenvironment. The prepared nanoparticles showed the conversion of APAP to the toxic metabolite NAPQ1, leading to GSH depletion and accelerating the effect of the Fenton reaction [83]. Zhong et al. prepared pH-responsive nanoparticles using BSA-derived albumin as carrier nanoparticles and encapsulating triphenylphosphine-modified DOX, which could be used to target tumor mitochondria [84]. Meng et al. prepared a metal-phenolic network-based multifunctional nanocomposite coated with Fe–tannic acid complexes and reported that Fe–tannic acid was degraded by laser irradiation (808 nm) and the acidic pH of the tumor environment, resulting in drug release and the Fenton reaction, promoting the effect of tannic acid [85]. Lei et al. prepared pH-responsive nanoparticles co-encapsulated with DOX and APAP, which were released at 56.5% and 61.8%, respectively, under an acidic endosomal/lysosomal environment, synergistically promoting OH^•^ generation by the Fenton reaction [86]. Cho et al. prepared dual (pH- and redox-)responsive magnetic nanoparticles that promote drug release under low pH and high GSH concentrations [87], and Chen et al. developed a pH/ROS-responsive multifunctional nanoplatform that inhibits tumor through chemo/photodynamic/chemodynamic combinations [88]. Jia et al. prepared multifunctional nanoparticles with a core-shell structure encapsulating Fe_3_O_4_ and demonstrated that simultaneous photothermal and chemodynamic therapy is possible [89]. In addition to tannic acid, several food components have been used as effective applications for anticancer therapy as follows: the generation of ROS by vitamin C based on the Fenton reaction of Fe_3_O_4_ nanoparticles in cells [90]; promotion of lipid peroxidation and induction of ferroptosis in anaplastic thyroid carcinoma produced by vitamin C via the Fenton reaction [91]; enhancement of linomycin release by the Fenton reaction using tea polyphenols [92].

In addition to Fenton reactions, approaches utilizing the Fenton-like reaction have also been utilized. Cheng et al. reported that the Cu^2+^ and polymersome complex efficiently induced the Fenton-like reaction and promoted the oxidation of iminoboronates [93]. Wang et al. reported that conjugation of nanoparticles composed of glucose oxidase, Cu_2_-xSe, and a membrane of 4T1 cells promoted the Fenton reaction by increasing H_2_O_2_ under NIR-II irradiation [94]. Sun et al. prepared nanotubes composed of SiO and Cu, which is advantageous for the combination of photodynamic therapy and photothermal therapy (PTT). The prepared nanotubes effectively promoted the generation of ROS by the reaction between Cu^2+^ with H_2_O_2_ in the Fenton-like reaction, PTT effect, and porous structure of the nanotubes [95].

## 5. Fenton Reaction in Food

It is known that complex interactions occur between metal ions (or protein–metal ion complexes) and food components. Research on the relationship between the Fenton reaction and food components is relatively advanced in terms of flavonoids. Flavonoids are known to have antioxidative effects and are regarded as candidates that modulate the Fenton reaction. Among the flavonoids, the antioxidant/prooxidant properties of luteolin or kaempferol in Fenton-like reactions have been reported. For example, it has been reported that coordination of luteolin or kaempferol to Cu(II) significantly suppresses the generation of hydroxyl and superoxide radicals by 80% in the Fenton-like reaction [96,97]. These Cu-flavonoid complexes are considered to have intercalation activity towards DNA, which have potential applications for disorders associated with oxidative damage. Perron et al. measured the oxidation rate of Fe^2+^ when several polyphenol compounds were bound and found that galloyl groups oxidize iron faster than catechol groups, suggesting that a single iron-binding moiety contributes to the protective effects of polyphenols against oxidative damage [98]. Proteins are also affected by the Fenton reaction at their amino acid residues; cysteine and methionine residues are especially easily oxidized [99,100]. Bochi et al. investigated the effects of Fenton reaction-generated advanced oxidation protein products on the gene transcription in HEK293 cells [101]. As a result, it activated the gene transcription of inflammatory genes (NF-κB, COX-2, and IL-6), possibly mediating inflammation in the kidneys. Ishikawa et al. reported that phosphoprotein phosvitin, known as iron-career in egg yolk, chelated iron more effectively than other iron-binding proteins such as ferritin and transferrin, and accelerated the oxidation of Fe^2+^ to inhibit the Fenton reaction [102]. In some cases, the Fenton reaction may play a role in improving food quality as an effective tool. Voltea et al. used the Fenton reaction to accelerate the oxidative brewing of white wines, enabling rapid testing to assess the susceptibility, appropriate levels of flavanols and total free sulfhydryls for subsequent processes [103]. Gharib-Bibalan et al. showed that the oxidation process via the Fenton reaction modified the color and total polyphenols, improving the quality indexes of the purified juice [104]. Blank et al. reported that the Fenton-type reaction has significant effects on the aroma of coffee beverages [105]. Yeung et al. hydrolyzed okra pectin by the Fenton reaction to obtain pectic oligosaccharides with low molecular weights (1.79–6.09 kDa) and improved bioactivity (antioxidant and anti-inflammatory) [106]. Food components should also interact with metal ions in the body, but there are few reports on this.

One of the most important concerns regarding commercial food is their safety. As the Fenton reaction generates strong toxic radicals, it is used to kill bacteria that cause food poisoning. Shi et al. developed the Fenton reaction-assisted photodynamic inactivation method, a simple system that combines calcinated melamine sponges and Fe^2+^ to inactivate Salmonella under light illumination [107]. Morikawa et al. developed two “green” iron catalysts with reducing and chelating ability using tea leaves and coffee grounds [108]. This system with the catalytic Fenton reaction enhanced the degradation of the contaminants into harmless compounds and disinfection of Escherichia coli. In contrast, the Fenton reaction can also work as a protective system for certain microbes. Calhoun et al. reported that Dps, a ferritin-like protein with DNA-binding properties, protects Salmonella enterica serotype Enteritidis against the common killing mechanism of bactericidal antibiotics through the Fenton reaction [109]. Since oxidation leads to food deterioration, the monitoring of food conditions is essential and several approaches using the Fenton reaction have been reported. Abbas et al. developed a simple and highly sensitive fluorometric method based on the Fenton reaction system to assess H_2_O_2_ in foods [110]. Additionally, Wang et al. developed a novel colorimetric and fluorescent ELISA based on the Fenton reaction triggered by glucose oxidation was constructed to quantitatively and qualitatively measure danofloxacin in milk [111].

## 6. Conclusions

This review summarized the Fenton reaction from the basic principle to the bioavailability of iron, and the latest applications in the medical and nutritional fields. In the medical fields, it appears that various nanomedicines utilize the intracellular Fenton reaction as the generating system of strongly toxic OH^•^ to enhance their selectivity and efficiency. In the nutritional fields, the Fenton reaction has been used to kill microorganisms that cause food spoilage, and this redox system has also been applied to food processing. The Fenton reaction is also useful in the synthesis of bioethanol [112,113] and the removal of pollutants derived from drugs or food additives [114,115,116,117]. Through this review, it can be inferred that the Fenton reaction can be used as a useful technology in both the medical and nutritional fields, though the mechanism is partially unknown. Additionally, the biosafety of Fenton-reaction-based nanomedicines is insufficient and unclear. Most papers regard the Fenton reaction as being useful, with less side effects than other drugs because the reaction is regulated by H_2_O_2_ and pH. However, for example, hypoxia, related to H_2_O_2_ generation, is a typical feature of solid tumors of cancer. Further investigation into the biosafety of Fenton-reaction-based treatment is warranted. It is expected that new technologies utilizing the Fenton reaction will continue to be developed.

On the contrary, it was found that the Fenton reaction of absorbed food components has been little examined to date. For example, vitamin C is one of the well-known antioxidants in the body, but it can also act as a pro-oxidant through the Fenton reaction [118]. Simultaneously, vitamin C changes into its oxidized form, dehydroascorbic acid. The mechanism by which high-level vitamin C kills cancer cells has been the subject of much debate [119], and recent studies have described the potential contribution of dehydroascorbic acid to cancer cell destruction [120,121]. This indicates the free-iron and Fenton reaction are involved in the functions of compounds with reduction properties, but their interaction has rarely been examined. As the redox system is too complicated in the body system, a comprehensive understanding might be necessary to elucidate the rules of their bioactivities. As science and technology advance in general, there will be a demand for a more sufficient understanding of the effects of these food components and Fenton reactions. More progress is expected in the near future.

## Data Availability

Not applicable.
